# mRNA vaccine: a potential therapeutic strategy

**DOI:** 10.1186/s12943-021-01311-z

**Published:** 2021-02-16

**Authors:** Yang Wang, Ziqi Zhang, Jingwen Luo, Xuejiao Han, Yuquan Wei, Xiawei Wei

**Affiliations:** Laboratory of Aging Research and Cancer Drug Target, State Key Laboratory of Biotherapy, National Clinical Research Center for Geriatrics, West China Hospital, Sichuan University, No. 17, Block 3, Southern Renmin Road, Chengdu, Sichuan 610041 PR China

**Keywords:** mRNA vaccine, Self-amplifying RNA, Non-replicating mRNA, Modification, Immunogenicity, Delivery strategy, COVID-19 mRNA vaccine, Clinical trials, Antibody-dependent enhancement, Dendritic cell targeting

## Abstract

mRNA vaccines have tremendous potential to fight against cancer and viral diseases due to superiorities in safety, efficacy and industrial production. In recent decades, we have witnessed the development of different kinds of mRNAs by sequence optimization to overcome the disadvantage of excessive mRNA immunogenicity, instability and inefficiency. Based on the immunological study, mRNA vaccines are coupled with immunologic adjuvant and various delivery strategies. Except for sequence optimization, the assistance of mRNA-delivering strategies is another method to stabilize mRNAs and improve their efficacy. The understanding of increasing the antigen reactiveness gains insight into mRNA-induced innate immunity and adaptive immunity without antibody-dependent enhancement activity. Therefore, to address the problem, scientists further exploited carrier-based mRNA vaccines (lipid-based delivery, polymer-based delivery, peptide-based delivery, virus-like replicon particle and cationic nanoemulsion), naked mRNA vaccines and dendritic cells-based mRNA vaccines. The article will discuss the molecular biology of mRNA vaccines and underlying anti-virus and anti-tumor mechanisms, with an introduction of their immunological phenomena, delivery strategies, their importance on Corona Virus Disease 2019 (COVID-19) and related clinical trials against cancer and viral diseases. Finally, we will discuss the challenge of mRNA vaccines against bacterial and parasitic diseases.

## Introduction

A vaccine stimulates the immune response of the body’s immune system to produce antibodies. Classical vaccine originates from anti-viral immunity [1]. Dating back to 1796, Edward Jenner found that healthy individuals inoculated with cowpox on the milkmaids’ hands had preventative immunity against smallpox infection [2]. Multiple virus-oriented vaccines are currently used for routine vaccination, which gains significant progress in preventing and treating viral diseases. Therefore, scientists are seeking to develop effective cancer vaccines. In 2006, the FDA approved the first cancer vaccine in human history: A vaccination against cervical cancer (Gardasil). Gardasil prevents the infection of human papillomavirus (HPV) 16/18 for more than 5 years, decreasing cervical cancer incidence [3, 4]. However, most of the cancer vaccines are under preclinical and clinical trials. Although much progress towards developing vaccines has been achieved, there still exist viral pathogens escaping the adaptive immune responses [5]. Besides, the increasing need for large-scale production and rapid development urges to develop novel vaccine approaches. Non-viral diseases, including cancer, need more vaccine-related researches to foster a novel vaccine development platform.

mRNA vaccine is a newly developed technology with a combination of molecular biology and immunology. The technology is closely related to gene therapy. The foreign mRNAs encoding antigens are introduced into somatic cells to synthesize antigens by the expression system [6]. The synthetic antigens can induce the immune response [7]. As early as the year 1990, scientists used mRNA expression vectors to inject mRNAs into mouse somatic cells in vivo to express luciferase, beta-galactosidase and chloramphenicol acetyltransferase [8]. In 1992, Jirikowski et al. found that adding mRNAs encoding oxytocin and vasopressin in diabetes insipidus mice (genetically mutant) reversed diabetes insipidus temporarily within several hours after injection [9]. Although remarkable findings had been achieved from then on, we made no substantive progress on mRNA studies. The challenges were mRNA instability, excessive immunogenicity and lack of effective mRNA delivery system [10–13].

During these decades, further researches and the improvement of experimental techniques have made progress in the safety, efficacy and industrial production of mRNA vaccines. These advantages enable mRNA-based vaccines a priority in the treatment of tumors and viral diseases. Firstly, mRNA vaccines are safe to induce antibodies in human phase I clinical trials [14]. The explanation is that mRNA is not a replicating vector. mRNA vector has no characteristics of antibiotic resistance, genomic integration and strong immunogenic responses [15–17]. Additionally, nucleases rapidly degrade single-stranded RNA [18]. Although the degraded mRNA components trigger the immune system’s excessive activation, developing an effective and safe delivery system with modified mRNA can enhance the efficacy and eliminate the side effects [19, 20]. Next, the improved therapeutic efficacy is realized by modified mRNAs and mRNA carriers. In viral diseases, the HSV-2 (herpes simplex virus 2) nucleoside-modified mRNA vaccine decreased virus loads [21]. Mannose-modified liposomes were used to deliver mRNA into cells. The vector protected mRNA from degradation and promoted mRNA overexpression by upregulating mannose receptor (CD206) on cell surfaces [22]. So far, various forms of delivery vectors and modified mRNAs have been deeply investigated to test their therapeutic efficacy [23], especially during the COVID-19 epidemic [24–27]. Finally, manufacturing mRNA vaccines on a large scale tends to be industrialized. The mass production-scale relies on translational science, which is critical to accelerate the production speed. In vitro, the translational technology rapidly selects formulations and constructs in preclinical and clinical studies [28].

Accumulated preclinical evidences are paving the way for future clinical evaluation. A series of clinical trials have been launched continually. Based on the rapidity of manufacture, the mRNA vaccine is a potential therapeutic method. In the review, we will first introduce the classification and the molecular features of mRNA vaccines. Then, the mechanisms increasing the antigen reactiveness would be discussed. We will also focus on the underlying anti-viral and anti-tumor mechanisms of mRNA vaccines in different delivery strategies to enhance the biotherapeutic efficacy. Furthermore, we will review the mRNA vaccine-related clinical trials, their immunological phenomena, delivery strategies, their importance on COVID-19 and related clinical trials against cancer and viral diseases. Finally, we will discuss why scientists do not choose mRNA vaccines against bacterial and parasitic diseases.

## Molecular biology of mRNA vaccines

### The classification and the structure of mRNA vaccines

mRNA is an intermediate product from transcription to translation, containing genetic information to guide corresponding proteins’ formation. The mRNA vaccine is a subtype of nucleic acid vaccines. It is divided into two categories: self-amplifying RNA (saRNA) and non-replicating mRNA. The conventional non-replicating mRNA is composed of a cap, 5′-untranslated regions (UTR), open reading frame (ORF) encoding vaccine antigens, 3′-UTRs and poly(A) tail. Except for ORF, other structural elements are crucial for the stability of mRNA and transcriptional efficiency. Those elements are also modifiable sites to prolong mRNA half-life in vivo and limit unwanted immune responses [29]. For example, a modified non-replicating mRNA encoding influenza H10 hemagglutinin (HA) induced type-I IFN-polarized innate immunity and vaccine-specific responses [30]. Moreover, the mRNA recruits a series of transcriptional factors to the cis-regulatory 5′-UTRs/3′-UTRs to control the translational speed and the half-life of mRNA [31, 32]. Every element plays an essential role in stabilizing the mRNA structure, controlling the accessibility to ribosomes and influencing the translational mechanisms [33, 34]. The modification of elements applies the conventional mRNA vaccines to clinical practice.

Compared with saRNA, convention mRNA vaccines are characterized by the small size, simple structure and the inclusion of only one ORF encoding vaccine antigens. One vaccine-antigen-specific ORF alone guarantees the absence of unwanted immune responses [10]. Although these characteristics seem to be propitious to conduct the preclinical investigation, scientists need to further prolong the period and increase mRNA expression levels in vivo.

**Fig. 1:**
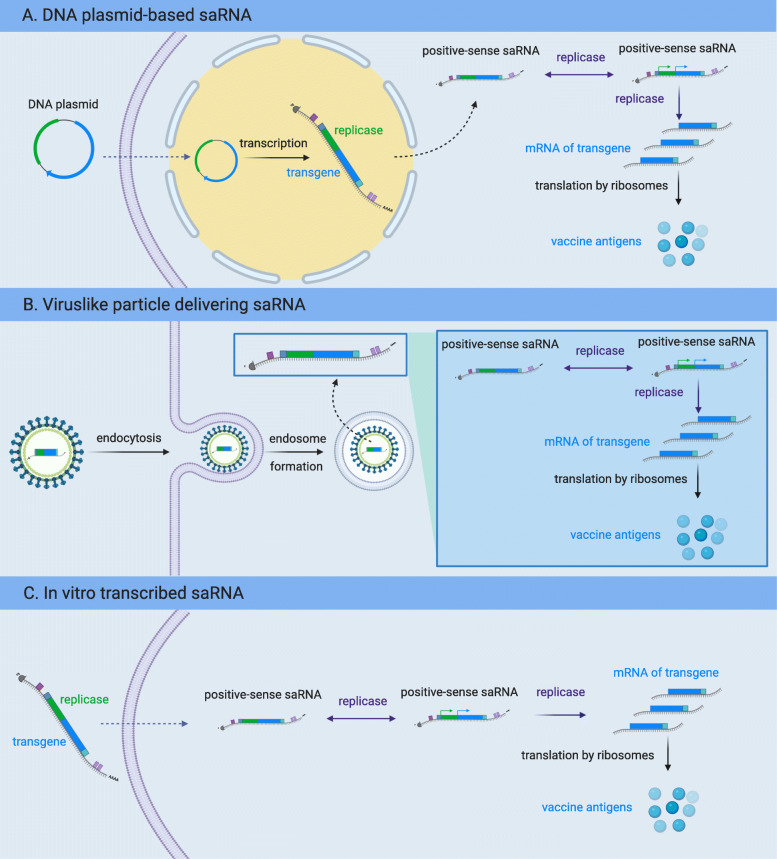
Antigen-encoding mRNA expression by alphaviral replicon RNA. **a** DNA plasmid–based saRNA uses plasmid DNA as a carrier to transfer replicase genes and the transgene into the nucleus where the mRNA is translated. **b** the virus-like particle packages saRNA and delivers replicon RNA to the cytosol by the receptor-mediated endocytosis, forming an endosome. **c** in vitro transcribed saRNAs are delivered in saline or synthetic formulations. Created with BioRender.com

Another classification of mRNA vaccines is saRNA. The saRNA vaccine is originated from the alphavirus genome. Such a vaccine comprises one gene responsible for the viral RNA replication and the other transgene encoding the therapeutic antigen [35]. According to different methods of obtaining antigen expression, the self-amplifying RNA embodies DNA plasmid-based saRNA, virus-like particle delivering saRNA and in vitro transcribed saRNA (shown as Fig. 1). Firstly, DNA plasmid-based saRNA uses plasmid DNA as a carrier to transfer replicase genes and the transgene into the nucleus. After the transcription in the nucleus, the replicon RNA unit (replicase and transgene) translocates to the cytosol for RNA self-replication, mRNA production and the translation of vaccine antigens. The advantages are the stability, simplicity of manufacture [36] and the induction of more powerful immune responses due to the stable DNA plasmid with higher levels of antigen expression [37]. Secondly, the virus-like particle packages saRNA and delivers replicon RNA to the cytosol. The particle recognizes the receptor on the cellular membrane. The receptor-mediated endocytosis generates endosomes in the cytoplasm. The following RNA self-replication processes to produce vaccine antigens are the same as DNA plasmid-based self-amplifying RNA. The virus-like particle has been proven to be safe and efficacious in clinical trials [38]. Finally, in vitro transcribed saRNAs are delivered in saline or synthetic formulations. Lipid nanoparticles or similar formulations are needed as vectors. If the formulation is fully synthetic, we should obey the enzymatic Current Good Manufacturing Practices (CGMP) process [39].

**Fig. 2:**
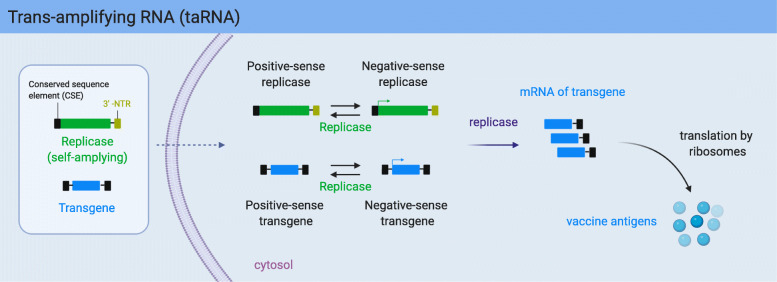
Trans-amplifying RNA (taRNA) The replicase transcribes a negative strand RNA with the 3′-nontranslated region (NTR). In turn, it uses the negative strand RNA as a template to transcribe a positive strand RNA from the 5′-NTR region. A promoter (arrow) initiates transcription into mRNA. Vaccine antigens come from the mRNA, which is mediated by cytoplasmic ribosomes. Created with BioRender.com

Based on the three kinds of saRNA structures, Beissert et al. developed an improved saRNA vaccine (trans-amplifying RNA (taRNA)) strategy to induce protective immunity (shown in Fig. 2). The taRNA relied on a bipartite vector composed of one RNA encoding the replicase and the other alphaviral RNA encoding vaccine antigens. The latter RNA did not contain the replicase to form a transreplicon with the former replicase-encoding RNA [40]. Beissert et al. used influenza hemagglutinin antigen-encoding RNA as an antigen RNA. A nanogram dose (50 ng) could elicit neutralizing antibodies and induce protective immune responses [40]. This novel taRNA excels at safety, manufacturability and ease of optimization. As for safety, the alphaviral RNA encoding vaccine antigens is separated from the whole. The separation refrains the saRNA from expressing viral glycoprotein, which helps saRNA transfer into other cells. Concerning manufacturability, long RNA transcripts do not limit the scaled-up production any more in taRNA, because the taRNA strategy shortens RNA lengths. Finally, ease of optimization can be realized by nucleoside modifications, codon optimization and stabilizing sequences [40]. Overall, the taRNA vaccine is a supreme strategy than conventional non-replicating mRNA vaccines.

### Eliminating immunogenicity of mRNA

Synthesizing mRNA by in vitro transcription (IVT) is not expensive [41]. However, the most challenging problem encountered by IVT mRNA is its immunogenicity. Exogenous IVT mRNAs are recognized by retinoic acid-inducible gene I (RIG-I) receptors, initiating innate immune responses [42]. IVT mRNA can activate immune cells and produce Toll-like receptor-mediated inflammation. The U-rich sequence of mRNA is a key element to activate Toll-like receptors [43]. By shortening the U-rich sequence, Thess et al. believe that it is a possible method to avoid mRNA immunogenicity [44].

Modifying nucleotides chemically, adding poly (A) tails and optimization mRNA with GC-rich sequence are effective methods to reduce the immunogenicity of mRNAs. First, nucleotide chemical modification does not influence the translation of mRNAs. Numerous scientists replaced cytidine with 5-methylcytidine (m5C), replaced uridine with 5-methyluridine (m5U), replaced adenosine with N1-methyladenosine (m1A) and N6-methyladenosine (m6A), 2-thiouridine (s2U), 5-methoxyuridine (5moU), pseudouridine (ψ) and N1-methylpseudouridine (m1ψ) [45]. Among them, m5C and ψ are preferable in base-pair modifications because they simultaneously reduce the immunogenicity and enhance the translation efficiency. Next, adding poly (A) tails decreases U content and shields mRNA in the sequence, thus lowering the mRNA immunogenicity [46]. Then, CureVac and Acuitas Therapeutics delivered erythropoietin (EPO)-encoding mRNA, which has rich GC codons, to pigs with lipid nanoparticles (LNPs). Their results indicated EPO-related responses were elicited without immunogenicity [44]. However, scientists should also consider the low efficacy of protein expression caused by excessive GC content.

Followed by IVT, mRNA purification are essential to eliminate immunogenicity [47]. The purification includes high performance liquid chromatography (HPLC), anion exchange chromatography, affinity chromatography and size exclusion columns, aiming at removing truncated transcripts [45, 48]. A good illustration is that Pardi et al. designed HPLC purified and m1ψ modified mRNAs encoding anti-HIV-1 antibody and delivered the mRNA with LNP. The results showed that the systematically administered mRNA-LNP expressed protective antibodies, helping mice get rid of HIV-1 infection [49].

### The stability of mRNA vaccines by sequence optimization

The mRNA is vulnerable to degradation. Stabilizing the mRNA existence will guarantee the expression effect. Multiple factors are influencing their expression and stability in cells. For example, 5′-UTR/3′-UTR around the ORF increases the half-life and the expression levels of vaccine mRNA [50]. 5′ cap modified with locked nucleic acid (LNA)-modified dinucleotide stabilizes the mRNA. The m(7(LNA))G[5′]ppp[5′]G 3 cap analogue increases the translational efficacy [51]. Apart from various modification forms of 5′ cap, like 7-methylguanosine [52], capping the mRNA at the 5′ terminus with enzymes is more effective than various forms of cap analogs [53, 54]. The poly(A) tail is another mRNA-stabilizing element. Deleting the poly(A) site from mRNA makes mRNA unstable, compared with the intact gene [55]. It is reported that removing poly(A) with polynucleotide phosphorylase reduced the size of polysomes, the rate of peptide elongation and the number of translational rounds, respectively [56]. Therefore, the poly(A) tail is essential to maintain the stability of mRNA and successful translation [57]. Furthermore, selecting modified nucleotides and synonymous nucleotides to replace codons is another method. Although synonymous codons are unable to change the sequence of amino acids, those changes increase the mRNA stability. Sometimes, they affect the mRNA secondary structure and posttranslational modifications [58]. Additionally, increasing G:C proportion of the mRNA strengthens the mRNA stability [59]. Overall, 5′-UTR/3′-UTR, 5′ cap, the poly(A) tail, rare codon and G:C proportion are all optimizable sites for strengthening the mRNA stability.

As mentioned above, replacing the mRNA sequence with rare codons and introducing modified nucleotides are likely to change the mRNA structure, the translational accuracy [60] and the protein-folding mechinery [61]. These changes further influence the type, intensity and specificity of immune responses.

## Mechanisms of mRNA vaccine-mediated immunotherapy

Since optimized mRNAs are continually existing in the cytosol, mRNA vaccines are being applied in disease-related immunotherapy. mRNAs are translated into corresponding antigens after being inoculated into host cells, imitating virus-infection-like humoral immunity and cellular immunity [62, 63]. The nature of the corresponding antigens is an immune response-inducing antigen. The mRNA vaccine enhances the host’s anti-virus and anti-tumor effects by increasing T cells’ antigen reactiveness.

### Increasing the antigen reactiveness

Some heterologous genes’ expression products affect immune cells directly, promoting the growth and proliferation of immune cells. Hence, they can enhance the host’s anti-tumor and anti-viral ability. A good illustration is that scientists used ovalbumin (OVA) to transfect into tumor cells. Tateshita et al. regarded lipoplex-type mRNA as a vector to deliver unstable mRNA (in serum-containing medium) into bone marrow-derived dendritic cells (BMDCs). The results showed prominent OVA-specific cytotoxic T lymphocyte activity in vivo and antitumor effect by expressing the OVA protein [64]. Similarly, as for viral diseases, Joe et al. investigated whether intranodally administrated nucleoprotein mRNA vaccine could induce protective immunity. Nucleoprotein is a conserved virus protein. They found that the nucleoprotein mRNA vaccine lowered immune cells’ infiltration and increased the infiltrating proportion of monocytes, MHC II^+^ alveolar macrophages and T cells. The intensive response was protective [65]. The spike protein of severe acute respiratory syndrome coronavirus 2 (SARS-CoV-2) is prefusion-stabilized. Corbett et al. used it to produce an mRNA vaccine and assessed viral replication in nonhuman primates. The results indicated that the mRNA vaccine generated intensive SARS-CoV-2 neutralizing antibodies without any pathologic changes in the lungs [25].

Tumor cells may evade and survive through various mechanisms when encountering immune cells [66]. For example, MHC-I is expressed on the surface of nucleated cells. The molecule presents epitopes of the antigens processed by antigen-presenting cells (APCs) for the recognition of other immune cells. As for tumors, presenting tumor-associated antigens (TAAs) to T cell receptors (TCRs) by MHC-I is an initiator of CD8^+^-cytotoxic-T-lymphocyte (CTL) activation [67]. Therefore, the down-regulation of MHC-I on tumor cell surfaces helps tumor escape from the immunologic surveillance [68]. To improve the tumor’s immunogenicity, introducing mRNA encoding MHC-I and TAA [69, 70] into tumor cells enabled the up-regulation of MHC-I. Thus, immune cells recognized tumor cells and pathogens rapidly, which improved the therapeutic efficacy of mRNA vaccines by enhancing viral/tumoral immunogenicity.

In mRNA vaccination, B cell immunity is an important immunological component after the dendritic cell (DC) maturation and the induction of robust T cell responses [71]. Most of the antiviral vaccines induce protective antibody responses. Antibodies are produced from germinal centers (GCs) in B cell follicles of secondary lymphoid organs. Before maturation, B cells experience proliferation, somatic mutation and selection of high-affinity mutants in GCs with the help of T cells [72]. Subsequently, B cells receive intact antigens presented by DCs to generate an antibody response [73]. If the antigen availability is sustained during germinal center initiation, antibody responses to vaccination would be robust. Robust antibody responses drive the increase of antibody titers and B cell/T follicular helper cell responses in the germinal center [74]. T follicular helper cells must be activated to promote sustained neutralizing antibody responses. Viral infections reply on this cellular immunity because viruses can evade humoral immunity. This process strengthens the potency of intramuscularly and intradermally delivered mRNA-LNP vaccines [23, 75]. For example, mRNA vaccine encoding RSV fusion (F) caused potent T cell and B cell immune responses in mice [76]. The subcutaneous administration of influenza antigen-encoding mRNA complexed with LNPs and PEI generated T cell and B cell responses in mice [77, 78]. The interaction of GCs and T follicular helper cells remains to be elucidated. Understanding this process will facilitate future vaccine design.

### Induced innate immunity and adaptive immunity

mRNA vaccines induce innate immunity and adaptive immunity. Innate immunity is the first defensive line against non-self substances. The pathogen-associated molecular patterns (PAMPs) on mRNA is recognized by pattern recognition receptors (PRRs) on cell surfaces [79]. The binding of the ligand-receptor complex transduces signals into cells, further initiating a series of cascades of signaling pathways. Activated second messagers translocate to the nucleus as transcriptional factors, recruiting different trans-acting factors to promote the expression of proinflammatory cytokines and chemokines [80, 81]. Before the activation of adaptive immunity, it is essential to understand how cells sense non-self mRNA and initiate cascades of signaling pathways by the interaction of mRNA, PRRs and PAMPs. There are two kinds of PRRs that sense extracellular and intracellular PAMPs, respectively [82]. On the one hand, the recognition of RNA inside the endosome is Toll-like-receptor (TLR)-mediated [83]. Accumulated evidence shows that the TLR-MyD88-NFκB signaling pathway is regularly involved in PAMP recognition [83]. TLR-3 recognizes and binds to double-stranded RNA (dsRNA), modulating the activation of type I interferon (IFN) pathway and the secretion of cytokines and chemokines [84]. Alternatively, as a PAMP, single-stranded RNA (ssRNA) is combined with TLR-7 to activate nitric oxide synthase (NOS2) [85]. On the other hand, the cytosolic non-self RNA is recognized by RIG-I receptors [42], nucleotide oligomerization domain (NOD)-like receptors (NLRs) [86], RNA-dependent protein kinase receptor (PKR) [87] and oligoadenylate synthetase (OAS) receptors [88]. Activated RIG-I recognizes a novel long non-coding RNA (Lnczc3h7a), together with TRIM25 (an E3 ubiquitin ligase that mediates K63-linked ubiquitination of RIG-I), to strengthen RIG-I-mediated antiviral innate immunity [89]. Another RNA sensor (PKR) regulates the transcription factor IRF1, preventing the translational process from shutting down to fight against the virus [86]. Together, whatever RNA sensor is, RNA-induced PRRs contribute to type I IFN production. IFN-γ positively promotes the activation of PKP and the phosphorylation of eIF2α.

Nevertheless, at the same time, a negative feedback loop is formed to restrict the production of IFN-γ, affecting mRNA translation and posttranslational modifications [90]. Moreover, the overexpression of IFN promotes the binding of OAS and dsRNA for generating RNase L to degrade non-self RNA. Therefore, the optimized mRNA vaccines should meet the requirement that the innate immunity is fully activated to initiate the adaptive immunity. mRNA sequence designers should avoid excessive activation of the innate immunity that hinders mRNA translation.

### Immunological adjuvants

Co-administration of mRNA vaccines and their corresponding adjuvants can enhance the body’s immune response to antigens. Immunobiologically, the adjuvant is supplemented to enhance immunogenicity, increase titers of antibodies, alter antibody types and strengthen delayed hypersensitivities. However, the adjuvant mechanism is not completely clear and the mechanism of different adjuvant action is different. First, saRNA delivered by a cationic nanoemulsion (CNE) delivery system based on Novartis’s proprietary adjuvant MF59 is well-tolerated and immunogenic [91, 92]. TriMix is another mRNA adjuvant that includes three immune-modulatory molecules (active TLR-4, CD40 ligand and CD70). TriMix mRNA incorporated with other tumor-antigen mRNAs are administrated into stage III or IV melanoma patients. It showed augmented immunity and achieved a durable clinical relief [93]. In multiple vaccine studies, TriMix is involved in promoting DC maturation and CTL activation [94]. The preclinical studies have made TriMix towards clinical trials [95–97]. A third adjuvant is the RNActive (CureVac AG) vaccine platform containing both free and protamine-complexed mRNA. The vaccine combines properties of adequate antigen expression and autologous self-immune-stimulation well. The technique relies on the type of mRNA carrier, because it is responsible for providing adjuvant activity. Protamine is a crucial delivery element which has intrinsic adjuvanticity. It contributes to expressing vaccine antigens and stimulating innate immunity by the activation of TLR-7 [98]. In the study, inoculating the self-adjuvanted vaccine indicated a durable T cell-mediated immunity. In other words, the RNActive vaccine activates T cell-based immunity. T cells are transformed into antigen-specific memory T cells for the recognition of non-self antigens [99]. In human clinical trials, RNActive vaccines have good tolerability and immunogenicity [98, 100, 101]. RNAdjuvant is an innovative adjuvant whose nature is a 547-nucleotide non-coding ssRNA. A cationic peptide can stabilize poly U repeats in the ssRNA [102]. Mechanically, the RNAdjuvant induces neutralizing antibodies by TLR7-dependent activation of markers on DCs and the production of IFN-I. In MyD88−/−Cardif−/− mice, the lack of TLR and RIG-I-like helicase caused a reduced adjuvant effect [102]. In cancer patients, the RNAdjuvant upregulated CD80, CD86 and HLA-DR in circulating DCs, which promoted CD4^+^ T cell activation [103].

### mRNA vaccines elicit humoral immune responses without antibody-dependent enhancement (ADE) activity

ADE is a phenomenon that antibody protection against other viruses can deteriorate the infection and trigger harmful immunopathology [104]. Cross-reactive antibodies against the epitope on the E protein of zika virus deteriorate the dengue virus infection [105]. The phenomena should also be taken into consideration in developing coronavirus vaccines [106]. Therefore, ADE has been a significant concern for vaccine development.

Currently, Laczko et al. designed nucleoside-modified mRNA vaccines encapsulated with LNPs (mRNA-LNP). The mRNA encodes the full-length SARS-CoV-2 spike protein. The SARS-CoV-2 mRNA vaccine induced high levels of S protein-specific IgG. To further investigate whether or not the mRNA vaccine could elicit antibody-mediated ADE, they used HEK293T cells expressing mouse FcgR1. The results indicated no SARS-CoV-2 ADE by testing the mRNA-vaccinated mouse sera [107].

However, most animal models and in vitro models seldom predict ADE. One reason is that antibody-mediated mechanisms are the same. Another is that designing animal models depends on understanding how antiviral responses become harmful in humans [104]. Hence, we need more studies to define the clinical correlation with protective immunity. Moreover, we should carefully analyze the safety of mRNA vaccine in humans because ADE of diseases cannot be predicted after administrating antibodies and vaccination.

## Delivery strategies of mRNA vaccines

**Fig. 3:**
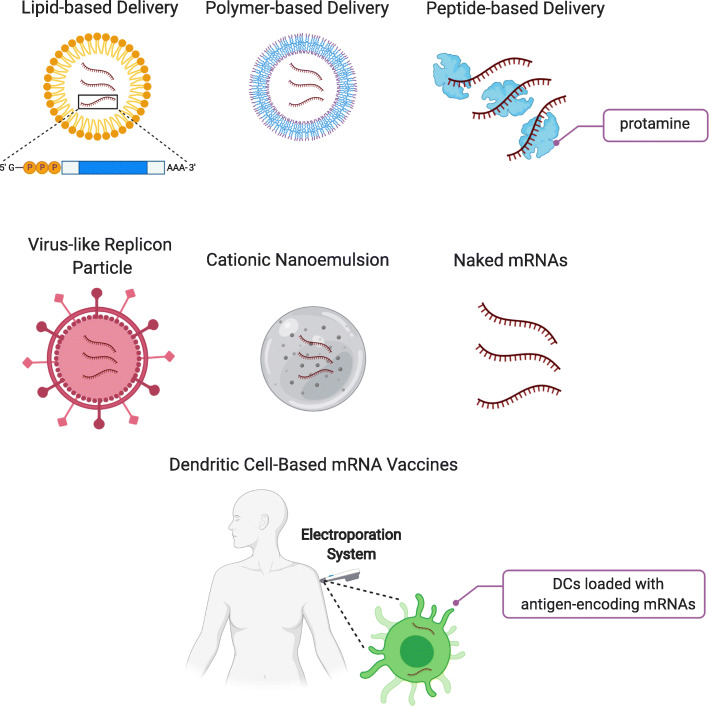
Major delivery methods for mRNA vaccines Commonly used delivery methods and carrier molecules for mRNA vaccines are shown: lipid-based delivery, polyer-based delivery, peptide-based delivery, virus-like replicon particle, cationic nanoemulsion, naked mRNAs and dendritic cell-based delivery. Created with BioRender.com

Due to the instability of mRNA vaccines, the introduction of mRNA vaccines needs some carriers’ assistance. Hence, scientists have developed lipid-based delivery, polymer-based delivery, peptide-based delivery, virus-like replicon particle delivery and cationic nanoemulsion delivery. Furthermore, the naked mRNA vaccine can also be directly injected into cells. To date, DC-based mRNA vaccines are newly developed to elicit adaptive immunity. This part will introduce the delivery strategies of mRNA vaccines from three aspects (every delivery method is shown in Fig. 3).

### Carrier-based mRNA vaccines

#### Lipid-based delivery

Lipid nanoparticles (LNPs) have been widely used as carriers to deliver mRNAs [27, 108, 109]. The first conducted clinical trial of mRNA vaccines is LNP-delivered. LNPs are mature negatively-charged nucleic acid delivery platforms characterized as ionizable amino lipids, polyethylene glycol, phospholipids and cholesterol. The essential ionizable amino lipids facilitate mRNA to escape from the endosome by interacting with ionizable amino lipids and the endosomal membrane [110]. Polyethylene glycol is another essential component to prolong LNP circulation time because it spatially hinders the binding of mRNA and plasma proteins, which accelerates the clearance by reticuloendothelial (ER). Phospholipids and cholesterol can stably integrate the LNP structure [111]. To be specific, LNPs have two advantages as an mRNA vaccine vector. On the one hand, LNPs defend mRNA from degradation by enzymes from the endosome [112]. The characteristic guarantees high encapsulation efficiency [113]. On the other hand, LNPs have good biocompatibility through a series of biological processes to deliver mRNAs for expression. The first process involves the apolipoprotein E (ApoE)-low density lipoprotein receptor (LDLR) pathway. This endogenous pathway is a targeting foundation for efficient delivery [113]. Then, the TLR4-mediated endocytosis takes up LNPs, forming a vesicle to fuse with endosomes [114]. After escaping endosomes, LNPs release mRNA into the cytoplasm to synthesize vaccine antigens.

The delivery efficacy of LNPs relies on multiple lipid components and lipid-related modifications. Foremost, the primary substance is cationic or ionizable lipids. Various cationic or ionizable lipids are used to deliver RNA, such as N-[1-(2,3-dioleoyloxy) propyl]-N,N,N-trimethylammonium chloride (DOTMA) [115], dilinoleylmethyl-4-dimethylaminobutyrate (Dlin-MC3-DMA), N,N-Dimethyl-2,3-bis[(9Z,12Z)-octadeca-9,12-dienyloxy]propan-1-amine (DLinDMA) [116], 1,2-dioleoyl-sn-glycerol-3-phosphoethanolamine (DOPE) [117], 1,2-dioleoyloxy-3-trimethylammonium propane chloride (DOTAP) [118] and N^1^,N^3^,N^5^-tris(3-(didodecylamino)propyl)benzene-1,3,5-tricarboxamide (TT3) [119]. These lipids are good delivery helpers because they are positively charged at a certain pH. Thus the negatively charged mRNA interacts with those lipids electrostatically for delivery. Due to cellular membrane structure, mRNA-encapsulating lipids easily fuse with the targeted cellular membrane [120]. Subsequently, the endocytosis of LNPs triggers proton-pump-mediated pH reduction. At this pH, the ionizable cationic lipid becomes more positively charged. Then, endogenous anionic lipids remove the cationic lipids by binding to the cationic lipids to generate a non-bilayer structure. Such a process results in disrupting the endosomal membrane and releasing mRNAs into the cytoplasm [121]. Secondly, the lipids’ head and tail are important modification sites for enhancing delivery efficacy. YSK12-C4 is a pH-sensitive cationic lipid. On the one hand, the hydrophilic head of YSK12-C4 determines the acid dissociation constant (pKa), an indicator of intrahepatically distributional conditions and the endosomal escape. On the other hand, the hydrophilic tail is a non-pKa-dependent structure and shares similar functions with the hydrophilic head [122]. Thirdly, Anderson et al. developed an isocyanide-containing lipid library. Isocyanide is a linker of di-hydroimidazole. They added di-hydroimidazole to LNPs for the optimization of mRNA delivery. Consistently, they added the STING (stimulator of interferon genes) agonist to LNPs for internalization. The adjuvant stimulation increases the mRNA efficiency. Additionally, they used LNPs to deliver antigen-specific mRNA vaccines in several mouse models for vaccination. The results showed an increased survival rate [123].

The administration methods determine the distribution and expression kinetics of lipid-based mRNA vaccines in vivo. Local delivery is realized by subcutaneous (SC) administration, intramuscular (IM) administration, intradermal (ID) administration and intranodal (IN) administration. Scientists applied these methods to APCs and other immune cells, eliciting locally strong and long-lasting immune responses. So local delivery is used to initiate the stimulatory reaction in the small area. For example, the lipid-polymer-RNA lipopolyplexes (LPRs) containing a tri-antenna of α-d-mannopyranoside (triMN-LPR) were administrated intradermally (ID) to C57BL/6 mice. The triMN-LPR increased the local inflammatory responses several days later and recruited the activated DCs to the lymph nodes around the intradermal injection site. When injecting triMN-LPR encapsulating vaccine antigens to tumor-bearing mouse models intradermally, Moignic et al. found a more robust stimulatory immune response to fight against cancer [124]. Another example is that mannosylated LNPs delivering influenza (hemagglutinin) encoded saRNA vaccine generated faster antigen-specific CD8^+^ T cell responses by intradermal administration [125]. Subcutaneous administration facilitated the PEGylated LNPs to be uptaken by the DCs in the lymph nodes and allowed the rapid release of mRNA vaccines after cellular internalization of LNPs [126]. Intravenous (IV) administration is a kind of systematic delivery. Compared with local delivery, intravenously administered mRNA antigen vaccines generate extensive and effective immunity [127]. IV administration causes the accumulation of mRNA-LNPs in the liver, resulting in the tremendous translational activity of proteins and the productive protein-synthesizing activity [128]. Immunologically, intravenously administered LNPs encapsulating mRNA antigen vaccines can mature DCs and activate antigen-specific T cells both in vivo and in vitro. When scientists intravenously immunized mice with OVA-mRNA-encapsulating LNPS, mice with lymphoma were presented with the inhibition of tumor growth and the recovery of abnormal hemogram [129]. Therefore, the IV administration of LNPs is commonly used in systematic diseases, like hematological diseases.

In short, the LNP is currently a potential mRNA-delivering candidate for good biocompatibility, high delivering efficacy and so on. Except for the above preclinical investigation, phase I (NCT04064905) and phase II clinical trials (NCT03897881) are undergoing for evaluating LNP-based mRNA antigen vaccines.

#### Polymer-based delivery

Polymeric materials are less clinically investigated as ionizable lipids do. However, they coat mRNA without suffering from degradation and promote protein expression. The disadvantages of polymeric materials are polydispersity and the clearance of large molecules [130]. To improve the therapeutic effect, scientists have added lipid chains, expand branch structures and construct biodegradation-promoting domains [131–133].

The classification of polymers contains the cationic polymer and the anionic polymer. As for the cationic polymer, polyethylenimine (PEI) [134], polyamidoamine (PAMAM) dendrimer [135] and polysaccharide [136] are the three members. Since saRNA is sensitive to the RNase and is taken up inefficiently by DCs, condensing mRNA into the PEI-polyplex vehicle tackles the problem. An mRNA encoding hemagglutinin of influenza virus and nucleocapsid are both coated with the PEI-polyplex. The cytosolic complex facilitates the mRNA translation and induces both humoral and cellular responses [78]. In another study, the polymer-based intranasal mRNA vaccination system is designed to overcome the difficulty that the nasal epithelium serves as a barrier to hinder delivering antigens to nasal associated lymphoid tissue (NALT). The cyclodextrin-polyethylenimine 2 k conjugate (CP 2 k) with HIV gp120 mRNA undoes tight junctions for paracellular delivery. Both the paracellular delivery and intracellular delivery can extend the residential time in the nose and initiate immense anti-HIV responses by producing cytokines and a balanced Th1/Th2/Th17 type [137]. Next, the dendrimer is a potential delivery vector because it has multiple functional groups with high tolerability. PAMAM dendritic polymers with NH_2_ and OH end functionalities respectively enter A549 human lung epithelial carcinoma cells faster than hyperbranched polymers [138]. PAMAM dendrimers were once used to construct antigen-encoding saRNA to protect mice from Toxoplasma gondii, Ebola and H1N1 influenza [139]. Taken mice with Zika virus for example, premembrane (prM)- and envelope (E)-encoding saRNA with dendrimer formulation enhanced IgG concentration and induced CD8^+^ T cell-dominating responses [140]. Subsequently, Shi  et al. used PAMAM (generation 0) dendrimer complexed with ceramide-PEG and poly(lactic-co-glycolic acid) (PLGA) to transfect phosphatase and tensin homolog (PTEN) mRNA for the restoration of tumor-growth suppression [141]. However, due to the spatial conformation of PAMAM, the biodegradation rate is limited, resulting in toxic accumulation. Hence, such a limitation hinders clinical development. Finally, chitosan is a polysaccharide substance involved in nanoparticulate delivery vehicles (nanogel-alginate (NGA)). Similarly, influenza virus hemagglutinin- and nucleoprotein-encoding mRNA are delivered to DCs by the chitosan-based nanoparticle. The vector promoted the successful translation of the two foreign antigen genes [77]. The delivery system favors the naked RNA passing through the cellular membrane and surviving in the biological condition.

Although cationic polymers are the dominant material, anionic ones are also sometimes used to deliver. The most commonly used anionic molecule is poly D, L-lactide-co-glycolide. Since the negatively charged mRNA is uneasy to be delivered by anionic polymers, the addition of cationic lipid in a PGLA complex would assist in establishing an efficient RNA-encapsulating system [142]. In recent years, PLGA-incorporating nanoparticles coated with LNPs have validated its delivery efficiency for up to 80%. The complex system rapidly induced the protein translation, reaching a peak in a short time and vanishing after 48 h. The mRNA characteristic is in accordance with such a phenomenon [143]. Consistently, researches from Sharifnia et al. [144] and Zhao et al. [145] both favor the advantage of the PLGA nanoparticle system. In lymphoma-bearing mouse models, lipid-assisted nanoparticles (CLAN) encapsulating ovalbumin (OVA) mRNA reduced the tumor growth rate [129].

Collectively, the polymer-based delivery system is a promising platform for its mRNA-delivering efficacy. However, the investigation is still in the early stage of preclinical trials. More problems need to be further illustrated.

#### Peptide-based delivery

Due to the electrostatic interaction, the negatively charged mRNA is easily delivered by the cationic peptide. The reason why peptides are positively charged is the positively charged amino groups. For example, the lysine residue and the arginine residue bring positive charges to the amino acid, enabling electronegative mRNA to adsorb onto the cationic peptide tightly [146]. The amount of loaded mRNA is positively correlated with N/P (negative/positive) ratios [147]. Additionally, an increased ratio of charged amino/ phosphate groups can increase zeta potential and minimize the particle size, increasing encapsulating efficiency [147].

Protamines are one of the cationic peptides to deliver mRNA. Two aspects make it a potential vector. On the one hand, protamines can protect mRNA from RNase-mediated degradation in the serum [148]. Stitz et al. used CureVac’s RNActive technology, in which the protamine-formulated RNA is an initiator of immunity. The protamine stabilized the immunogenicity at the changing temperature without affecting the antigen-encoding mRNA vaccine [149]. If not the RNActive vaccine platform, the protamine-formulated RNA alone would inhibit the translational process, further affecting the vaccine efficacy [150]. The reason for it is the excessively tight combination of the protamine and mRNAs [151]. On the other hand, protamine is an adjuvant. A study demonstrated that the protamine-formulated mRNA activated DCs and monocytes, secreting TNF-α and IFN-α. It also activated immune cells by the TLR-7/TLR-8-mediated recognition of the protamine-formulated mRNA. The protamine-formulated mRNA shared some structural similarities with condensed RNA in the nucleocapsids of RNA viruses [152]. Another study proved its antitumor priority over naked nucleic acid adjuvants in glioblastoma mouse models [153]. Clinically, of all the peptide-based carriers, only the protamine is undergoing evaluations [154–156].

Cationic cell-penetrating peptides (CPPs) are another kind of small peptides containing 8-30 amino acids. They are excellent delivery vehicles because they are not only equipped with low charge densities, but also able to disrupt membrane for endosomal escape. The latter reason is essential for synthesizing proteins [6, 147]. Coolen et al. compared the three-CPP mRNA platform, namely RALA (WEARLARALARALARHLARALARALRACEA), LAH4 (KKALLALALHHLAHLALHLALALKKA) and LAH4-L1 (KKALLAHALHLLALLALHLAHALKKA). The three peptide/mRNA complexes were all introduced into DCs and generated innate immunity. Mechanically, this process was PRR-mediated and fostered adaptive immune responses. Meanwhile, the uptake process and their intracellular activities involve clathrin-mediated endocytosis and phagocytosis. Among the three, the LAH4-L1/mRNA complex showed the optimal protein expression [157]. Another investigation combined the cationic characteristic and the cell-penetrating characteristic by formulating the fused protamine-CPP protein. The fused protein delivered reporting genes to human cell lines [158].

Like polymer-based delivery, anionic peptides are also conjugated to a positively charged substance because two negatively charged substances repel each other. For example, the addition of a positively charged copolymer p(HPMA-DMAE-co-PDTEMA-co-AzEMAm) (pHDPA) is to encapsulate the OVA-mRNA. Then, the azide groups on pHDPA conjugate an anionic peptide GALA (N-WEAALAEALAEALAEHLAEALAEALEALAA-OH-C). Such a formulated complex, as a vector, showed enhanced EGFP-mRNA transfection in RAW 246.7 macrophages and DCs. It entered DCs by sialic acid-mediated endocytosis, regardless of the maturation of DCs. Introducing GALA contributed to cell uptake and mRNA release from the endosome by integrating with the sialic acid group on the DC surface. The uptaking process is more efficient than the lipofectamine. Based on the effective transfection, the OVA-mRNA-encapsulating complex triggered prominent immune responses [159].

#### Virus-like replicon particle

Virus-like Replicon Particles (VRPs) can encapsulate antigen-encoding saRNA for delivering into the cytosol, which is like a virus-infecting manner. The viral structure proteins are synthesized in vitro, followed by encapsulating designated antigen-encoding saRNA. Some attenuated viruses maintain the ability of self-replication [160]. In multiple virus types, the enhancement of vaccine potency has been validated. Most recently, alphavirus-derived replicon RNA encoding the SARS-CoV-2 spike (S) protein has been encapsulated in lipid inorganic nanoparticles (LIONs). The formulated vaccine was intramuscularly injected into mice and primates, indicating the increased level of anti-SARS-CoV-2 S protein IgG antibody [161]. In another study, VRP was based on an HIV-derived mRNA encoding clade C envelope glycoprotein. A VRP packaged the mRNA. In rhesus macaques, the complex provoked cellular immune responses [91]. In Venezuelan equine encephalitis VRP, the mRNA encoded two kinds of E antigen of dengue virus (subviral particles [prME] and soluble E dimers [E85]). The immunization of such a VRP induced protective efficacy and E85-VRP had priority in speed and magnitude of immunity [162]. Another example is the flavivirus Kunjin based mRNA encoding granulocyte colony-stimulating factor (G-CSF). The VRP with the mRNA inhibited the growth of subcutaneous CT26 colon carcinoma and B16-OVA melanomas for half by inducing CD8^+^ T cells [163]. Lundstrom summarized viral saRNA replicon particles against viral diseases (Influenza, HIV, SIV, Ebola, Lassa, SARS-CoV, MERS-CoV, RSV, MPV, Dengue, HBV and CMV), bacterial diseases (*P. falciparum*, M. tuberculosis, C. botulinum, *B. abortus*, B. antracis, malaria, *L. monocytogenes*, prion and staphylococcus) and cancer, respectively [164].

Even if VRP had a therapeutic effect on viral diseases, bacterial diseases and cancer, there are two limitations. First, large-scale production has not been realized. The current time-consuming production process is limited by producing VRPs from cell lines [165]. The second one is that the complex would promote anti-vector antibodies’ production, impeding the ongoing clinical trials [166]. Hence, we should lower the immunogenicity of VRPs.

#### Cationic nanoemulsion

Cationic Nanoemulsion (CNE) is a non-viral delivery system, which potentiates the mRNA vaccines by binding to saRNAs. One of the most essential components is the cationic lipid 1,2-dioleoyl-sn-glycero-3-phosphocholine (DOTAP). DOTAP has been utilized in clinical trials for its positive charges emulsified with the same component of the emulsion adjuvant MF59. Except for clinical use, DOTAP has priority in availability, squalene solubility and the cationic feature at a certain pH [92]. Cationic lipids can form a pH-dependent nano-sized emulsion [167].

Brito et al. investigated CNE-delivered saRNA in animals (rabbit, mouse, nonhuman primate). Their saRNA encoded multiple vaccine antigens, such as the fusion (F) glycoprotein of respiratory syncytial virus (RSV), the envelope glycoprotein B (gB) of human cytomegalovirus (hCMV), a fusion protein (pp65-IE1) of phosphoprotein 65 (pp65) and immediate early protein 1 (IE-1) of hCMV and gp140 envelope glycoprotein (env) of the human immunodeficiency virus (HIV). The results demonstrated good CNE efficacy, the induction of immune responses by the adjuvant subunit and the low dose of CNE complex [92]. Consistently, it is validated that the cellular immune responses induced by saRNA-delivered CNE were more robust than that by saRNA-delivered VRP. The dose is as low as 50 μg, which is safe enough for immunogenicity [91]. Similarly, to fight against the venezuelan equine encephalitis virus and Zika virus, scientists developed the saRNA vaccine delivered by CNE, both inducing robust protective immunogenicity [168, 169].

Based on the above preclinical studies, CNE has the potentials in human clinical evaluations.

### Naked mRNA vaccines

Different from the carrier-based mRNA vaccines, naked mRNAs are delivered by directly injecting the mRNA solution. Although naked mRNAs cannot cross freely through the membrane, several studies proposed some hypotheses about its uptaking mechanism. Some researchers suggest that the uptake of naked mRNA involves DC-mediated macropinosytosis. It allows the expression of the antigen-encoding mRNA and promotes the T cell/DC activation. Once DCs are mature, mRNAs are diminished by DCs [170, 171]. Without the carrier’s assistance, other scientists believe that intracellular mRNAs are delivered by membrane disruption (direct penetration and permeabilization). Microinjection is a representative of direct penetration, which first began in the 1970s [172]. Permeabilization includes mechanical membrane disruption, electroporation [173], thermal membrane disruption, optoporation, biochemical membrane disruption and gated channels/valves [174].

The naked-mRNA solutions commonly used are Ringer’s solution and lactated Ringer’s solution [175, 176]. Both of them contain calcium, which is beneficial for mRNA uptake [177]. In several clinical trials, the two kinds of solutions were utilized. In the first trial, RNAs were dissolved in 1.0 mg/ml Ringer’s solution, followed by injecting into lymph nodes [178]. Next, the 80 μg mRNA vaccine was dissolved in Ringer’s lactate solution for the intradermal injection on the backs of C57BL/6 mice. Upregulated TLR7/8 drove the activation of immune cells, produced cytokines and activated innate and adaptive immunity [179].

The naked mRNA has a series of advantages. Firstly, mRNA would not be integrated into the genome. Then, cytosol-located ribosomes combine with mRNA directly, instead of DNA transfering from the nucleus to the cytosol. Thirdly, once mRNAs reach the cytosol, the translation process initiates immediately. This advantage determines the rapid immune responses after mRNA administration. Fourthly, the final location of mRNA determines the location of protein expression. Finally, mRNAs excel DNAs in decreasing toxicity and immunogenicity [174].

When it comes to its disadvantages, the vulnerability of RNase degradation may first come to our minds. However, the instability of mRNA in the serum can be compensated by changing administration methods and appropriate chemical modifications. To be specific, local delivery can avoid RNase interference from the blood, such as ID [179], IN [65], IM [76] and so on. However, most of the studies focus on the treatment of cancer.

#### Direct injection of mRNA cancer vaccines

Accompanied by different delivery methods (SC, IM, ID and so on), direct injection of naked mRNA is an efficient mRNA-delivering method. When it comes to cancer, intranodal delivery facilitates antigen delivery to APCs at the activated-T-cell site without recruiting DCs. A study demonstrated the optimization of intranodally administrated RNA vaccine in melanoma-bearing mice by systematically co-administrating DC-activating Fms-like tyrosine kinase 3 (FLT3) ligand, which is an adjuvant. Such a co-administration exerted improved CD8^+^ T cell priming and expansion in lymphoid organs, T-cell homing to melanoma and enhanced therapeutic activity of intranodally administrated RNA [180]. Moreover, another study investigated whether intranodal co-delivery of TAA mRNA with TriMix could mature DCs and further primeTAA-specific T cells, finding that CD11c^+^ cells in lymph nodes selectively uptaked and translated the mRNA. Meantime, the co-administration induced a stimulatory environment, generating CTL and therapeutic effects in multiple mouse models [181].

Intratumoral administration is another helpful method because it only rapidly activates tumor-related immunity, instead of introducing mRNA encoding tumor-associated antigens. Hewitt et al. found that intratumoral mouse (m)interleukin-12 mRNA therapy enhanced antitumor activity by anti-PD-L1. The regression of mIL-12-uninjected distal lesions was observed. Local injection of mIL-12 is proven to be a systematic effect [182]. Consistently, Jeught et al. constructed a fusion mRNA (IL-β and a domain of transforming growth factor-β receptor II). Intratumoral delivery of the mRNA had a therapeutic potential, which can be strengthened by blocking PD-1 and PD-L1 interactions [183]. As for TriMix mRNA, its intratumoral delivery produced a systematic antitumor immunity, which largely relied on tumor-infiltrating DCs (TiDC) [184].

Combining commonly used antitumor therapy with mRNA vaccines can improve vaccination outcomes. A good example is that a patient with melanoma receiving anti-PD-1 antibodies and neoepitope-encoding mRNA vaccines had higher efficacy [178]. Another illustration is adding a chemotherapy drug (cisplatin) with mRNA vaccines regressed tumors [94]. Nevertheless, the underlying mechanism remains to be elucidated.

### Dendritic cells-based mRNA vaccines

DC is an ideal vaccine target. The primary reason is that DCs, as an APC, internalize, process and present antigens to immune cells, which generates effective adaptive immunity [185]. To be specific, such an effect results from not only the upregulation of major histocompatibility complex (MHC) molecules for combining antigens [186], co-stimulators for providing secondary signals and various cytokines for T cell proliferation and the formation of CTL [187], but also the secretion of chemokines for T cell recruitment [188]. As early as in the 1990s, it is reported that DCs reliably primed T cells in situ. T cells recognized the MHC molecules from the original priming DCs [189]. The following three parts will discuss representative strategies for enhancing DC Targeting and Expression in DCs, the DC mRNA vaccines in inflammatory diseases and cancers.

#### Ex vivo and in situ loading strategy of engineered DCs with mRNA

DCs are loaded with mRNA both ex vivo and in situ. For the ex vivo condition, immature DCs are obtained from patients’ peripheral blood. After the maturation of DCs, DCs are loaded with antigen-encoding mRNA. Then, the engineered DCs are administrated back to patients.

Loading antigen-encoding mRNAs into DCs can be realized by electroporation, lipofection, nucleofection, and sonoporation ex vivo. Among them, the electroporation technique is the most frequently used [190–192]. Adding granulocyte-macrophage colony stimulating factor (GM-CSF) and IL-4 to DCs is a common method for DC differentiation [193]. GM-CSF attracts immune cells and molecules to the DC site as a stimulator of immunity, promoting antigen presentation. Clinical trials (NCT03396575 and NCT00204516) involves GM-CSF/mRNA-incorporating DC vaccines. Mature DCs express co-stimulatory molecules on their surfaces. The co-stimulatory molecular is one of the determinant factors to exert therapeutic efficacy [194]. Another factor is the ability of DCs to secret IL-12p70, which is an indicator of responses of DC vaccines [195]. This ability is strengthened by stimulating DCs with TLR ligands and proinflammatory cytokines [196].

For the in situ condition, DC transfection can be realized by directly injecting antigen-encoding mRNAs complexed with TriMix into lymph nodes. A clinical trial (NCT01684241) conducted an intranodal injection of naked mRNA in patients with advanced melanoma. TriMix showed priority in the stimulation of DCs and the enhancement of effector T cell functions, compared with other stimulatory cytokines [197]. NCT01066390 is the first clinical trial of TriMix-DC vaccine in patients with advanced melanoma. The combination of a checkpoint inhibitor (ipilimumab) and TriMix-DC vaccine showed satisfying results (NCT01302496).

#### Representative strategies of enhancing DC targeting and expression in DCs

DC targeting and mRNA expression in DCs are critical to the systematic administration of mRNA vaccines. One of the challenges is that the systematic administration of mRNA vaccines causes the aggregation of serum proteins and mRNA degradation. To overcome the difficulty, scientists developed various molecular carriers formulating mRNAs discussed above in detail. Such complexes contributed to mRNA uptake, enhance mRNA translational activities and protect it from degradation.

Another challenge is the systematically delivered biodistribution of mRNA vaccines. The issue is a huge obstacle of DC targeting after systematic administration. Pardi et al. attempted to deliver mRNA-LNPs intravenously and intraperitoneally by incorporating HPLC purified, 1-methylpseudouridine-containing mRNA and firefly luciferase into stable LNPs. The systematic delivery activated mRNA translation in the liver for several days [128]. In 2016, Kranz et al. developed an effective strategy for DC targeting after systematic administration. They believed that DCs were efficiently and precisely targeted in vivo by intravenous injection of RNA-lipoplexes (RNA-LPX). RNA-LPX is optimally net charge-adjusted and functional molecular ligand-free. A positively charged lipid particle targets the lung, while a negative one targets DCs in secondary lymphoid tissues and bone marrow. The LPX protects RNA from ribonuclease degradation, facilitates its efficient uptake and promotes the expression of the encoded antigen by DC populations and macrophages. Mechanically, the IFN-α secreted by DCs and macrophages matures DCs in situ and activates inflammatory immunity in the early stage of viral infection. mRNA-LPXs (endogenous encoding self-antigens, mutant neo-antigens and viral antigens) induced potent effector and memory T-cell responses. As for immune responses against tumor-specific antigens, they observed apparent tumor regression in multiple mouse models [198]. After being qualified in safety evaluation in mice and nonhuman primates, mRNA-LPXs undergo two clinical trials in patients with triple negative breast cancer and in patients with melanoma (NCT02316457 and NCT02410733).

#### DC mRNA vaccines in viral diseases

A well-known DC mRNA vaccine for viral diseases is the HIV-1 vaccine. Individuals infected with HIV-1 received DCs electroporated with multiple HIV-1 antigen-encoding mRNA vaccines. The cellular immune responses’ evaluation suggested antigen-specific T cell responses without clinical benefits [199–201]. Referring to electroporation, we often use it to introduce mRNA vaccines for its high mRNA delivery efficacy [202]. Mechanically, it disrupts the cellular membrane to allow the introduction of mRNA [203]. Parameters like voltage, electroporation solution, density, pulse time, cell number and RNA quantity can optimize the delivery efficiency [64, 204]. Even if former investigations have applied the mRNA electroporation to inflammatory diseases, most of the electroporation-related mRNA vaccine studies are about cancer.

#### DC mRNA vaccines in cancer

Based on the above features of DCs, DCs can also be utilized to deliver mRNA for cancer biotherapy, eliciting antigen-specific immune responses. In 1996, Boczkowski et al. were the first to discover that DCs pulsed with mRNA are a potential platform to elicit T cell responses. In this study, DCs pulsed with encoding-OVA mRNA (tumor-derived) were more effective in promoting OVA-specific CTL responses in vitro than DCs pulsed with OVA peptide. In vivo, a principal reduction of metastatic lung sites was witnessed in tumor-bearing mouse models (B16/F10.9) with poor immunogenicity and massive metastases when the mice received the OVA-mRNA vaccine [205]. Apart from OVA, other immunity-regulating proteins have been investigated in the form of mRNA. These protein-encoding mRNA served as an adjuvant to improve the efficacy of DC-based mRNA vaccines. The electroporation of DCs with mRNA encoding 4-1BB ligand (4-1BBL) [206], CD83 [207], tumour necrosis factor receptor superfamily member 4 (TNFRSF4) [208], p53 [209] and CD133 [210] all primed anti-tumor CTLs. Furthermore, proinflammatory cytokines play a role in modulating DC functions, such as GM-CSF [70], IL-12p70 [211], IL-12 and IL-18 [212]. Besides, TriMix can be electroporated with the addition of mRNAs. For example, mRNA encoding Wilms’ tumor gene 1 (WT1), survivin and TriMix can be electroporated [213]. In a study, patients received ipilimumab (IPI) and DCs electroporated with mRNA encoding TriMix and tumor-associated antigens tyrosinase (gp100, MAGE-A3 and MAGE-C2). Robust CD8^+^ T cell responses and clinical responses were witnessed in stage III/IV melanoma patients [93]. Consistently, Lint et al. found that TriMix stimulated antitumor T-cell responses [184]. The underlying mechanism is DC activation and the transition from T regulatory cells to T helper 1 (TH1)-like cells [214, 215]. A phase IB clinical trial indicated that melanoma patients were administrated with DCs electroporated with mRNA encoding TriMix and tumor-associated antigen had prolonged progression-free survival time and tolerance [216].

## The COVID-19 mRNA vaccines

We further emphasize the importance of the mRNA vaccine in the Corona Virus Disease 2019 (COVID-19). At the end of 2019, the epidemic of COVID-19 began to emerge due to the Severe Acute Respiratory Syndrome Coronavirus 2 (SARS-CoV-2) virus. Since then, multiple vaccine-developing researches had been conducted [24, 26, 107, 217–220].

### The efficacy of mRNA vaccines against SARS-CoV-2

The successful preclinical investigation and clinical investigation proved the antigen-encoding mRNA vaccine to be effective and significant. In the preclinical study, the administration of mRNA encoding SARS-CoV-2 virus-like particles in mice was proven to generate a robust antiviral-like immune response [107, 218]. Consistently, Zhang et al. encapsulated mRNA encoding the receptor-binding domain (RBD) of SARS-CoV-2 with a lipid nanoparticle. They injected such a formulated vaccine into mice and nonhuman primates intramuscularly, inducing specific neutralizing antibodies and Th1-biased cellular response [219]. Subsequently, a vaccine for clinical trials was urgently developed. One of the well-known vaccines is BNT162b1, a lipid nanoparticle-formulated mRNA vaccine encoding SARS-CoV-2 spike glycoprotein RBD. Local delivery of BNT162b1 is dose-dependent. The RBD-specific IgG and SARS-CoV-2 neutralizing titers increased after a second injection [221, 222]. Based on the curative effects, the phage I/II/III clinical investigation totally recruited 29,481 participants (NCT04368728).

### The advantages of mRNA vaccines against SARS-CoV-2 over other kinds of vaccines

mRNA vaccines are promising candidates against SARS-CoV-2, compared with other kinds of vaccines (inactivated vaccines, attenuated live vaccines, passive immunization-related vaccines, subunit vaccines, synthetic peptide vaccines, recombinant antigen vaccines, DNA vaccines and so on). Unlike DNA vaccine, mRNA vaccinated do not enter the nucleus to participate in DNA structural transformation. So the antigen-expressing mechanism is simpler and safer. Compared with traditional vaccines, mRNA vaccine design needs virus gene sequences, instead of virus strains. The production of mRNA vaccines does not need cell culture or animal matrix, which means that the production process is simpler than protein and that the cost is lower. Meanwhile, mRNAs are a component of human sapiens cells. They can be naturally degraded with no metabolic toxicity. If SARS-CoV-2 mutates, it is much easier to modify the mRNA sequence than modify the protein structure. More importantly, the outbreak pandemic requires us to shorten the period of vaccine researches. The period of developing mRNA vaccine is shorter than inactivated vaccines, attenuated live vaccines and subunit vaccines [223].

### The pandemic of COVID-19 gives opportunities to mRNA vaccine development

However, mRNA vaccines were less attractive before the twenty-first century. The reason is summarized in two aspects. On the one hand, mRNAs are not easy to be modified. They are not stable as DNA and proteins and they are vulnerable to be degraded. On the other hand, they induce more robust immune responses like virus invasion. So there are substantial safety risks to mRNA vaccines. Until 2005, Professor Katalin Kariko and Drew Weissman in the University of Pennsylvania had made a breakthrough [46]. They discovered that the key to induce mRNA-mediated immune responses was a nucleotide (uracil). It escaped the surveillance of the immune system when it was modified as pseudouracil. After solving the most crucial issue (safety), developing mRNA vaccines would become more rapid. During these years, mRNA vaccines have evolved in synthesis, modification, delivery and production. Until the epidemic of COVID-19, mRNA vaccines exerted more public focus for their safety, efficacy and industrial production.

## Clinical trials of mRNA vaccines

The antigen-encoding mRNA vaccine is a promising candidate for its safety, the improved therapeutic efficacy and a large scale mRNA-vaccine production. The disadvantages of mRNA are compensated by multiple delivery systems in a series of viral and cancerous preclinical studies. Therefore, developing cancer- and viral disease-oriented mRNA vaccine is worth numerous dedication. We summarized important clinical trials of mRNA vaccines against cancer (shown as Table 1) and viral diseases (shown as Table 2) in different delivery strategies, respectively. As for tumors, most of clinical trials focus on melanoma, glioblastoma, prostate cancer and leukemia. As for viral diseases, the majority of clinical trials is about SARS-CoV-2 and HIV-1.

**Table 1:** Clinical trials with mRNA vaccines against cancer

Cancer tpye	NCT number	Drug administration	Phase	Status
Non-small Cell Lung Cancer	NCT03164772	BI 1361849 (CV9202) + Durvalumab+/−Tremelimumab	I/II	Recruiting
	NCT03908671	Personalized mRNA vaccine encoding neoantigen	–	Not yet recruiting
	NCT02688686	Suppressor of cytokine signaling (SOCS) 1, MUC1 and Survivin mRNA-loaded DC + cytokine-induced killer	I/II	Unknown
Ovarian Cancer	NCT04163094	W_ova1 + carboplatin/paclitaxel	I	Recruiting
	NCT01334047	DC-006 vaccine	I/II	Terminated
	NCT01456065	DCs loaded with TERT-mRNA and Survivin-peptide	I	Unknown
Melanoma	NCT00204607	mRNA+GM-CSF	I/II	Completed
	NCT00978913	DCs transfected with hTERT, survivin and p53	I	Completed
	NCT00940004	Dendritic cells electroporated with mRNA encoding gp100 and tyrosinase	I/II	Completed
	NCT01066390	TriMix-DC	I	Completed
	NCT02285413	DCs loaded with mRNA encoding tumor-associated antigens gp100 and tyrosinase+/−cisplatinum	II	Completed
	NCT00204516	mRNA coding for melanoma associated antigens+GM-CSF	I/II	Completed
	NCT01278940	mRNA-transfected DCs + IL-2	I/II	Completed
	NCT01530698	autologous dendritic cell vaccine by mRNA Electroporation	I/II	Completed
	NCT00243529	Autologous dendritic cell vaccine	I/II	Completed
	NCT03897881	mRNA-4157 + pembrolizumab	II	Recruiting
	NCT01456104	Autologous Langerhans-type dendritic cells electroporated with mRNA encoding a tumor-associated antigen	I	Active, not recruiting
	NCT02410733	Lipo-MERIT	I	Active, not recruiting
	NCT00961844	Dendritic cells - transfected with hTERT-, survivin- and tumor cell derived mRNA+ex vivo T cell expansion and reinfusion+Temozolomide	I/II	Terminated
	NCT03480152	(NCI)-4650, a mRNA-based, personalized cancer vaccine	I	Terminated
	NCT00929019	Autologous dendritic cells electroporated with mRNA	I/II	Terminated
Brain Cance (mainly glioblastoma)	NCT00846456	Tumor stem cell derived mRNA- transfected dendritic cells	I/II	Completed
	NCT00626483	CMV pp65-LAMP mRNA-loaded DC + GM-CSF	I	Completed
	NCT03548571	DCs transfected with mRNA encoding survivin and hTERT+temozolomide	II/III	Completed
	NCT03927222	Human CMV pp65-LAMP mRNA-pulsed autologous DCs + temozolomide+tetanus-diphtheria toxoid+GM-CSF	II	Recruiting
	NCT02649582	Autologous WT1 mRNA-loaded DC + temozolomide	I/II	Recruiting
	NCT03688178	Human CMV pp65-LAMP mRNA-pulsed autologous DCs + temozolomide+varlilumab+tetanus-diphtheria (Td) toxoid+111In-labeled DCs + unpulsed DCs	II	Recruiting
	NCT02465268	pp65-shLAMP mRNA DCs + GM-CSF	II	Recruiting
	NCT02808416	Personalized cellular vaccine	I	Active, not recruiting
	NCT02709616	mRNA-TAA pulsed autologous DC	I	Active, not recruiting
	NCT00639639	CMV-ALT+CMV pp65-LAMP mRNA-loaded DC (CMV-DC)	I	Active, not recruiting
	NCT02366728	Human CMV pp65-LAMP mRNA-pulsed autologous DCs	II	Active, not recruiting
	NCT01291420	WT1 mRNA-electroporated autologous dendritic cell	I/II	Unknown
	NCT00890032	BTSC mRNA-loaded DCs	I	Unknown
Prostate Cancer	NCT01278914	mRNA-transfected dendritic cells	I/II	Completed
	NCT01446731	DCs transfected with PSA, PAP, survivin and hTERT mRNA+docetaxel	II	Completed
	NCT02692976	DC loaded with protamine/mRNA encoding keyhole limpet hemocyanin (KLH) + DC loading with MHC I binding peptides, NY-ESO-1 and MUC1 PepTivator®	II	Completed
	NCT01197625	Dendritic cell vaccine	I/II	Active, not recruiting
	NCT01153113	Human telomerase reverse transcriptase mRNA (hTERT mRNA) transfected dendritic cell	I/II	Withdrawn
	NCT02140138	CV9104	II	Terminated
	NCT02452307	Peptide vaccine+montanide ISA-51+/−GM-CSF+/−imiquimod+/−mRNA/protamin	I/II	Unknown
Blood System Cancer (leukemia mainly)	NCT00834002	Wilms Tumor Gene (WT1) mRNA-transfected autologous dendritic cell	I	Completed
	NCT01734304	DCs electroporated with mRNA encoding WT1, PRAME, and CMVpp65	I/II	Completed
	NCT00510133	GRNVAC1 (mRNA encoding human telomerase reverse transcriptase (hTERT) and a portion of the lysosome-associated membrane protein LAMP-1 (LAMP))	II	Completed
	NCT02528682	MiHA mRNA-loaded PD-L-silenced DC	I/II	Completed
	NCT01686334	Autologous WT1 mRNA-electroporated DCs	II	Recruiting
	NCT01995708	CT7, MAGE-A3, and WT1 mRNA-electroporated Langerhans cells (LCs)	I	Active, not recruiting
	NCT03083054	Autologous dendritic cells electroporated with WT1 mRNA	I/II	Active, not recruiting
	NCT00514189	Autologous dendritic cells	I	Terminated
	NCT00965224	mRNA coding for Wilms’ tumor antigen WT1	II	Unknown
Digestive System Cancer	NCT00228189	CEA mRNA-loaded DCs	I/II	Completed
	NCT03468244	Personalized mRNA vaccine encoding neoantigen	–	Recruiting
	NCT02693236	Adenovirus-transfected autologous DCs + CIK cells	I/II	Unknown

**Table 2:** Clinical trials with mRNA vaccines against viral diseases

Infectious disease tpye/ Virus type	NCT number	Drug administration	Phase	Status
SARS-CoV-2	NCT04523571	BNT162b1 + placebo	I	Recruiting
	NCT04449276	CVnCoV Vaccine+placebo	I	Recruiting
	NCT04470427	mRNA-1273 + placebo	III	Recruiting
	NCT04368728	BNT162b1 + BNT162b2	I/II/III	Recruiting
	NCT04515147	CVnCoV	IIA	Not yet recruiting
	NCT04283461	mRNA-1273	I	Active, not recruiting
	NCT04405076	mRNA-1273 + placebo	IIA	Active, not recruiting
Rabies	NCT02241135	CV7201 mRNA encoding the rabies virus glycoprotein	I	Completed
	NCT03713086	Rabipur®	I	Active, not recruiting
HIV-1 Infection	NCT00833781	mRNA-transfected autologous DCs+/− autologous DCs with no mRNA transfection	I/II	Completed
	NCT02413645	TriMix mRNA+/−HIV mRNA	I	Completed
	NCT02888756	iHIVARNA-01 + TriMix+/−Placebo	IIA	Terminated
Zika Virus	NCT03014089	mRNA-1325 + placebo	I	Completed
	NCT04064905	mRNA-1893 + placebo	I	Active, not recruiting
Tuberculosis	NCT01669096	GSK 692342	II	Completed
Human Metapneumovirus and Human Parainfluenza Infection	NCT03392389	mRNA-1653 + placebo	I	Completed
	NCT04144348	mRNA-1653 + placebo	Ib	Recruiting
Ebola Virus Disease	NCT02485912	two separate mRNAs encoding two Zaire strain Ebola glycoproteins, respectively	I	Completed
Influenza	NCT03076385	VAL-506440 + placebo	I	Completed
Respiratory Syncytial Virus	NCT04528719	mRNA-1345 + placebo	I	Not yet recruiting
Cytomegalovirus Infection	NCT03382405	mRNA-1647, mRNA-1443	I	Active, not recruiting
	NCT04232280	mRNA-1647 + placebo	II	Active, not recruiting

## Discussion and conclusion

In the past few decades, we witnessed the efficiency of mRNA vaccines in different delivery strategies, due to the safety, efficacy and industrial production of mRNA vaccines. In 2020, mRNA vaccines exerted a public concern for COVID-19. The majority of studies aimed at treating cancer, such as melanoma. The rest of the studies focus on viral diseases, including COVID-19, rabies, respiratory syncytial virus infection, zika virus infection, cytomegalovirus infection, human metapneumovirus and human parainfluenza infection, HIV infection, Ebola virus infection and influenza.

Investigations of mRNA vaccines should not leave eliminating mRNA immunogenicity, stabilizing mRNA vaccines, increasing the antigen reactiveness, producing effective imminity, adding immunological adjuvants and developing effective delivery system. After sequence optimization, mRNAs are stable and less immunogenitic. Based on the immunology, mRNAs are first sensed by innate immunity, followed by PRRs/PAMPs-mediated cascades of the signaling pathway. Activated innate immunity triggers adaptive immunity. More extensive researches focus on different delivery strategies. LNPs, polymers and peptides have enabled the mRNA-delivering efficacy more robust and the exploration in-depth. Induced cellular responses and neutralizing antibodies are witnessed in mice, nonhuman primates and human beings. VRP and CNE also potentiate the delivering efficacy and broaden the scope of delivery strategies. Some immunologic adjuvants are also supplemented to enhance immunogenicity, increase titers of antibodies, alter types of antibody production and strengthen delayed hypersensitivities. Alternatively, the naked mRNA showed the priority under the condition of appropriate administration methods and chemical modifications. Furthermore, the classical APCs (DCs) can deliver antigen-encoding mRNAs by encapsulation, eliciting antigen-specific immune responses.

Most of the mRNA vaccine studies are tumoral and viral. Except for tumoral and viral targets, scientists also applied mRNA vaccines to bacterial infection and parasitic diseases. For example, Maruggi et al. used saRNA encoding antigens from Group A and Group B Streptococci to immunize mice with Group A Streptococci infection and Group B Streptococci infection separately. The mRNA vaccine had sustained protection in mice by producing functional serum antibodies [224]. Another study evaluated saRNA efficacy in malaria. saRNA encoding plasmodium macrophage migration inhibitory factor (PMIF) elicited cellular/humoral immune responses and PMIF-specific immunoglobulin G. The saRNA vaccine delayed blood-stage latency after sporozoite infection and increased the number of liver-resident CD8 + T cells and antigen-experienced memory CD4 + T cells. Surprisingly, the vaccine protected mice from re-infection by adoptively transferring CD8^+^ and CD4^+^ T cells [225].

Even so, researches of mRNA vaccines against bacteria and parasites are limited. No vaccine relating to bacteria and parasites has been approved so far. As for bacteria, they are one hundred times larger than viruses. Hence, there are differences in the structural complexity and immunology between them. The virus composition constitutes only dozens of antigens. However, bacterial composition can constitue thousands of antigens bacause bacteria have cell walls, cell membranes, fimbriae, capsules, proteins and nucleic acids. It is extremely difficult to find antigens that can be made into vaccines in bacteria. A second reason is that common bacterial diseases are not violent infectious diseases. Most diseases have been treated with effective antibiotics. The cost of antibiotic production is low. So mRNA vaccines are less important in bacterial diseases. As for parasites, it is difficult to obtain live attenuated vaccines in vitro because of their parasitism. Compared with bacteria, it is much more difficult to obtain effective and multivalent vaccines because parasites’ reproduction cycle and antigen composition are more complex. Moreover, some parasites induce severe hypersensitivity. Other parasites cause mechanical damage to host tissue and capture nutrition without causing immune responses. A proportion of parasites are able to escape immunity. More importantly, there have been effective antiparasitic drugs. Therefore, mRNA vaccines are less common in parasitic diseases.

In terms of the above difficulties, we should identify new candidate antigens and develop multiple multivalent vaccines. Most significantly, we should enhance vaccine’s immunogenicity and titers of protective antibodies.

Although mRNA vaccines in tumors and viruses have multiple advantages, they are still in the initial stage. Currently, safety is the most significant issue. ADE should be considered in developing mRNA vaccines. Their intended use would be assessed by cost/benefit ratio. Expenses and efforts determine the ultimate demonstration of solving a medical problem. We hope that mRNA vaccines’ future is bright. Clinical trials would turn basic reseach into mRNA therapeutics in medical practices.

## Data Availability

Not applicable.

## Abbreviations

HPV: Human papillomavirus
HSV-2: Herpes simplex virus 2
saRNA: Self-amplifying RNA
UTR: Untranslated regions
ORF: Open reading frame
HA: Hemagglutinin
CGMP: Current Good Manufacturing Practices
taRNA: Trans-amplifying RNA
LNA: Locked nucleic acid
TAAs: Tumor-associated antigens
TCR: T cell receptors
CTL: Cytotoxic-T-lymphocyte
OVA: Ovalbumin
BMDCs: Bone marrow-derived dendritic cells
SARS-CoV-2: Severe acute respiratory syndrome coronavirus 2
PRRs: Pattern recognition receptors
TLR: Toll-like-receptor
dsRNA: Double-stranded RNA
ssRNA: Single-stranded RNA
NOS2: Nitric oxide synthase
RIG-I: Retinoic acid-inducible gene I
NOD: Nucleotide oligomerization domain
NLRs: NOD-like receptors
PKR: Protein kinase receptor
OAS: Oligoadenylate synthetase
CNE: Cationic nanoemulsion
LNPs: Lipid nanoparticles
ER: Reticuloendothelial
ApoE: Apolipoprotein E
LDLR: Low density lipoprotein receptor
DOTMA: N-[1-(2,3-dioleoyloxy) propyl]-N,N,N-trimethylammonium chloride
Dlin-MC3-DMA: Dilinoleylmethyl-4-dimethylaminobutyrate
DLinDMA: N,N-Dimethyl-2,3-bis[(9Z,12Z)-octadeca-9,12-dienyloxy]propan-1-amine
DOPE: 1,2-dioleoyl-sn-glycerol-3-phosphoethanolamine
DOTAP: 1,2-dioleoyloxy-3-trimethylammonium propane chloride
TT3: N^1^,N^3^,N^5^-tris(3-(didodecylamino)propyl)benzene-1,3,5-tricarboxamide
STING: Stimulator of interferon genes
SC: Subcutaneous
IM: Intramuscular
ID: Intradermal
IN: Intranodal
APC: Antigen-presenting cells
LPRs: Lipid-polymer-RNA lipopolyplexes
IV: Intravenous
PEI: Polyethylenimine
PAMAM: Polyamidoamine
NALT: Nasal associated lymphoid tissue
CP 2 k: Cyclodextrin-polyethylenimine 2 k conjugate
PLGA: Poly(lactic-co-glycolic acid)
NGA: Nanogel-alginate
CLAN: Lipid-assisted nanoparticles
CPP: Cationic cell-penetrating peptides
VRPs: Virus-like Replicon Particles
LIONs: Lipid inorganic nanoparticles
G-CSF: Granulocyte colony-stimulating factor
CNE: Cationic Nanoemulsion
RSV: Respiratory syncytial virus
hCMV: Human cytomegalovirus
HIV: Human immunodeficiency virus
FLT3: Fms-like tyrosine kinase 3
TiDC: Tumor-infiltrating dendritic cell
MHC: Major histocompatibility complex
TNFRSF4: Tumour necrosis factor receptor superfamily member 4
WT1: Tumor gene 1
COVID-19: Corona Virus Disease 2019
SARS-CoV-2: Severe Acute Respiratory Syndrome Coronavirus 2
RBD: Receptor-binding domain
GCs: Germinal centres
IVT: In vitro transcription
EPO: Erythropoietin
ADE: Antibody-dependent enhancement
